# Integrating network, sequence and functional features using machine learning approaches towards identification of novel Alzheimer genes

**DOI:** 10.1186/s12864-016-3108-1

**Published:** 2016-10-18

**Authors:** Salma Jamal, Sukriti Goyal, Asheesh Shanker, Abhinav Grover

**Affiliations:** 1 School of Biotechnology, Jawaharlal Nehru University, New Delhi, 110067 India; 2 Department of Bioscience and Biotechnology, Banasthali University, Tonk, Rajasthan 304022 India; 3 Bioinformatics Programme, Centre for Biological Sciences, Central University of South Bihar, BIT Campus, Patna, Bihar India

**Keywords:** Alzheimer-associated genes, Machine learning, Interaction networks, Sequence features, Functional annotations, Molecular docking, Molecular dynamics

## Abstract

**Background:**

Alzheimer’s disease (AD) is a complex progressive neurodegenerative disorder commonly characterized by short term memory loss. Presently no effective therapeutic treatments exist that can completely cure this disease. The cause of Alzheimer’s is still unclear, however one of the other major factors involved in AD pathogenesis are the genetic factors and around 70 % risk of the disease is assumed to be due to the large number of genes involved. Although genetic association studies have revealed a number of potential AD susceptibility genes, there still exists a need for identification of unidentified AD-associated genes and therapeutic targets to have better understanding of the disease-causing mechanisms of Alzheimer’s towards development of effective AD therapeutics.

**Results:**

In the present study, we have used machine learning approach to identify candidate AD associated genes by integrating topological properties of the genes from the protein-protein interaction networks, sequence features and functional annotations. We also used molecular docking approach and screened already known anti-Alzheimer drugs against the novel predicted probable targets of AD and observed that an investigational drug, AL-108, had high affinity for majority of the possible therapeutic targets. Furthermore, we performed molecular dynamics simulations and MM/GBSA calculations on the docked complexes to validate our preliminary findings.

**Conclusions:**

To the best of our knowledge, this is the first comprehensive study of its kind for identification of putative Alzheimer-associated genes using machine learning approaches and we propose that such computational studies can improve our understanding on the core etiology of AD which could lead to the development of effective anti-Alzheimer drugs.

**Electronic supplementary material:**

The online version of this article (doi:10.1186/s12864-016-3108-1) contains supplementary material, which is available to authorized users.

## Background

Alzheimer’s disease (AD) is the most common neurological disease, accounting for 60–70 % of total dementia cases, affecting masses of people across the globe [1]. The growing incidences of this irreversible brain disease is due to lack of the effective treatment options, with the currently available drugs being able only to slow down the disease advancement and not halt it [2]. The neurodegenerative AD is characterized by short-term memory loss, challenges in completing daily activities, bafflement, problems in speaking and writing, changes in behavior and mood swings [3]. The socio-economic burden including medical expenses, costs associated with fulltime caregiving, etc. linked to the disease is huge which makes the disease as one of the most costly diseases [4]. Various hypothesis have been suggested to describe the cause of the disease, that include amyloid hypothesis, cholinergic hypothesis, tau hypothesis and genetic factors, yet the mechanism of the disease is poorly understood [5]. It has been proposed that genetic factors are mainly responsible for AD cases, and thus there have been many studies in quest for the genes associated with the disease and the unexplored principal genetic mechanisms [6].

A wide range of population surveys, genetic linkage studies and genome-wide association studies (GWAS) have been conducted to identify AD-associated genes and genetic mutations that alter with the expression of the genes in the brain. Apolipoprotein E (ApoE), Presenilin-1 (PSEN1) and Presenilin-2 (PSEN2), amyloid precursor protein (APP) and the linked mutations are some of the strongest risk factors that were observed to be associated with the brain disorder, Alzheimer’s [7]. Researchers have proposed that alteration of the functions of any of these genes results in enhanced production of amyloid beta peptide (Aβ) in the brain, extracellular aggregation of which leads to loss of synaptic functions and neuronal cell death resulting in AD. Several other genes that showed significant association with AD include sortilin-related receptor: L, clusterin, bone marrow stromal cell antigen 1, leucine –rich repeat kinase 2, complement receptor 1, phosphatidylinositol binding clatherin assembly protein 1 and Triggering receptor expressed on myeloid cells 2 and more [8]. A lot of other genes have been put forward through traditional methods of gene discovery like GWAS in populations and linkage studies, however owing to the time and labor consumed and the high risk rate, there appears the need for the methods which could significantly reduce the size of the candidate gene sets for genetic mapping [9]. Recently, a number of alternative approaches, like genomics, proteomics, bioinformatics and many other computational methods have been employed to identify the putative disease genes, mainly for cancer [10–12], decreasing the number of genes for experimental analysis.

Since the already discovered AD-associated genes do not cover a significant portion of the human genome, there can be an innumerable number of disease genes still left to be discovered. Thus, in spite of the discovery of many genes responsible for AD, identification of disease-associated genes in humans still remains a huge problem to be addressed. Additionally due to the fact that no cure for AD exists, the identification of novel AD genes can disclose novel effective therapeutic targets which could advance the discovery of drugs for the disease [2]. Lately, network-based methods integrating properties from protein-protein interaction (PPI) networks, have been widely used for prioritization of disease genes and finding an association between the genes and the diseases. Liu and Xie, 2013 integrated network properties from PPI networks, and sequence and functional properties and generated a predictive classifier to identify cancer-associated genes [13]. Vanunu et al. [14] also proposed a global network-based approach, PRINCE, which could prioritize genes and protein complexes for a specific disease of interest and applied the method to prioritize genes for prostate cancer, AD and type-2 diabetes mellitus.

In the present study, we have used machine learning approaches to generate highly accurate predictive classifiers which could predict the probable Alzheimer-associated genes from a large pool of the total genes available on the Entrez gene database. We have investigated the interaction patterns of the genes from their network properties using PPI datasets, and the sequence features and the functional annotations of the genes and employed these properties to classify disease and non-disease genes. We have used eleven machine learning algorithms and trained the classifiers using Alzheimer (Alz) and non-Alzheimer (NonAlz) genes and examined the relevance of the features in the classification task and studied their behavior for both the classes of the genes. Finally, to identify candidate drugs for the predicted novel genes we have used molecular docking approach and screened the already known approved and investigational Alzheimer specific drugs against the novel targets. To validate our initial findings and to further evaluate the affinity of the drugs against the predicted novel targets we have carried out molecular dynamics (MD) simulations and MM/GBSA calculations on the ligand-bound protein complexes. Using the computational approach presented in the current study, we have identified 13 novel potential Alz-associated genes which could prove beneficial for the development of drugs and improve our understanding of the AD pathogenesis.

## Methods

### Dataset source: positive and negative datasets

A total of 56405 genes belonging to *Homo sapiens* species were obtained from the Entrez Gene [15] database at the National Centre for Biotechnology Information (NCBI). Entrez Gene is an online database that incorporates extensive gene-specific information for a broad range of species, the information may comprise of nomenclature, genomic context, phenotypes, interactions, links to pathways for BioSystems, data about markers, homology, and protein information, etc. The positive dataset, Alz (AD-associated) consisted of 458 genes which had been reported as disease genes that could cause AD. All the other 55947 Entrez genes, excluding the AD-associated genes, were considered as NonAlz (not related to AD) genes which comprised the negative dataset.

### Mining biological features

#### Network features

To compute topological features of the Alz and NonAlz genes, human protein-protein interaction (PPI) datasets were retrieved from Online Predicted Human Interaction Database (OPID) [16], STRING [17], MINT [18], BIND [19] and InTAct [20] databases. We calculated 9 topological properties of the PPI network for each gene: the average shortest path length, betweenness centrality, closeness centrality, clustering coefficient, degree, eccentricity, neighborhood connectivity, topological coefficient and radiality (Additional file 1: Table S1). Average shortest path length or average distance is the measure of the efficiency of transfer of information between the proteins/nodes in a network through the shortest possible paths. Betweenness centrality, closeness centrality, eccentricity and radiality are the indicators of the centrality of a node in a biological network. Betweenness centrality and closeness centrality show the capability of a protein to bring together functionally relevant proteins and the degree of the transfer of information from a particular protein to other relevant proteins, respectively. Betweenness centrality is computed by totaling the shortest paths between the vertices passing through that node and closeness centrality is the sum total of the shortest paths between a node and all the other nodes. Eccentricity is the extent of the easiness with which other proteins of the network can communicate to the protein of interest. Radiality is the probability of the significance of a protein for other proteins in the network. Degree may be defined as the number of edges connected to a node while clustering coefficient is the degree of the nodes that tend to cluster together in a network. Neighborhood connectivity is a derivative of the connectivity; connectivity is the number of the neighbors of a node while neighborhood connectivity is the average of all the neighborhood connectivities. Topological coefficient is the extent of sharing of a node’s neighbors with the other nodes in the network. All the interaction datasets were loaded and integrated into Cytoscape [21], which is an open-source platform for visualizing molecular interaction networks, and Network Analyzer [22] plugin of Cytoscape was used for computing the topological parameters of the networks for 383 Alz and 13699 NonAlz genes.

#### Sequence features

UniProtKB (Universal Protein Resource Knowledgebase) [23], a freely accessible database which stores large amount of information on protein sequence and function, was used to obtain protein sequences corresponding to Alz and NonAlz genes. The protein sequence properties were calculated using Pepstats [24] program available from Emboss [25] and 21 sequence properties were extracted. The sequence features are molecular weight, the number of amino acid residues, average residue weight, charge, isoelectric point, molar extinction coefficient (A280), the frequency of the amino acids (Alanine, Phenylalanine, Leucine, Asparagine, Proline, Arginine, Threonine and Serine) and the amino acids grouped as polar and non-polar, small, aliphatic and aromatic, and acidic and basic (Additional file 1: Table S1). Only the reviewed protein sequences were considered for calculating protein sequence statistics, thus we retrieved protein sequences and calculated properties for 383 Alz and 13666 NonAlz genes.

#### Functional features

Using DAVID (Database for Annotation, Visualization and Integrated Discovery) [26], functional properties associated with the 370 Alz and 13549 NonAlz genes were incorporated. DAVID is an open-source knowledgebase by which one can obtain Gene Ontology (GO) terms for large gene lists. Two additional Swiss-Prot functional annotation terms, UP_SEQ_FEATURE and SP_PIR_KEYWORDS, were also included for the Alz- and NonAlz-associated genes. The number of genes (the Count term) linked to each functional annotation term was computed and only those terms were selected which had Count >38 i.e. associated with at least 1 % of the input Alz-associated genes. Further, the functional annotation terms were filtered based on *p*-value <0.001 and fold-enrichment >1.5 and the final 62 functional features were retrieved for the Alz and NonAlz genes. A list of final 62 functional features associated with the Alz and NonAlz genes has been provided as Additional file 1: Table S1.

### Feature selection

We employed feature selection techniques, to identify significant features contributing efficiently towards predicting the target class and thus extract the smaller subset of features for classification of Alz and NonAlz genes. Seven feature selection techniques were used that include a gain-ratio based attribute evaluation, oneR algorithm, chi-square based selection, correlation-based selection, information gain-based attribute evaluation and relief-based selection, to select the important attributes. Gain-ratio based attribute selection approach measures the gain ratio regarding the prediction class [27] while info-gain attribute evaluation [28] uses Info Gain Attribute Evaluator and measures the information gain with respect to the prediction class. Chi-squared Attribute Evaluator calculates the chi-square statistic with respect to the class. OneR [29] algorithm uses OneR classifier for attribute selection and generates one rule for each attribute followed by selecting the attribute with smallest-error to be used for classification. Correlation-based selection employs CfsSubsetEval and measures the worth of a subset of attributes by evaluating each predictor [30]. The algorithm finally selects the subset in which the predictors are highly correlated with the prediction class while are poorly correlated to other predictors. Relief-based selection evaluates the importance of an attribute by choosing the instances randomly and considering the value of an attribute for the nearest neighboring instance [31]. Weka [32], a publicly available machine learning software, was used for implementing the above mentioned feature selection algorithms for the purpose of selection of meaningful attributes.

Additionally, Principal Component Analysis (PCA) was conducted using FactoMineR [33] package available from R platform. The first two principal components explained around 60 % of the variance (Additional file 2: Figure S1) and attributes having >0.1 value of loadings in PC1 and PC2 were retained. The attributes selected by 5 out of the 7 selection methods and had >0.1 value of loadings in PCA were considered for training the model systems for Alz and NonAlz genes predictions.

After the extraction of relevant features, the combined positive and negative datasets were split into 80 % training set and 20 % test set using ‘create Data Partition’ function available from CARET [34] package of R.

### Machine learning based model systems generation

Eleven machine learning algorithms were applied to generate classifiers using the training dataset which could predict Alz- and NonAlz-associated genes using the selected network, sequence and functional features [35]. The machine learning methods used include Naive Bayes (NB) [36], NB Tree [37], Bayes Net [38], Decision table/Naive Bayes (DTNB) hybrid classifier [39], Random Forest (RF) [40], J48 [41], Functional Tree [42], Locally Weighted Learning (LWL (J48 + KNN(k-nearest neighbor)) [43], Logistic Regression [44] and Support Vector Machine (SVM) [45]. SVM model using Radial Basis Function (RBF) kernel was generated using the CARET package of R. Weka package was used to build all the other classifier models. Default parameter settings were used for generating all the classifier models.

Ten-fold cross-validation was used for training the classifier models to overcome the problems of overfitting of the generated models and to gain insights into the performance of the models on independent test sets. In cross-validation, say k-fold cross-validation, the training data was split into k subsets or folds and the models were generated using k-1 subsets and the remaining one set was used as previously unseen test set for the generated models. This process was repeated until all the k folds were used as test set at least once. The cross-validation results reported are the averaged over all the generated training classifier models.

### Cost-sensitive classifier

In order to remove bias in classification of the positive and negative datasets, misclassification costs were applied to the classifiers. Costs were introduced through a 2X2 confusion matrix which was divided into true positives (TP), false positives (FP), true negatives (TN) and false negatives (FN). The costs were applied on FN and a total of 22 classifier models were generated which include 11 models generated using base classifiers and 11 cost-sensitive models [46, 47].

### Performance assessment of generated classifier models

The performance of the generated 11 cost-sensitive classifiers in classifying Alz and NonAlz genes was measured using accuracy, precision, recall, F-measure or F1score and Matthews Correlation Coefficient (MCC). Accuracy (TP + TN/(TP + TN + FP + FN)) is proportion of the correct positive and negative classifications by the classifier models. Precision (TP/(TP + FP)) is the percentage of true positives while recall or sensitivity or TP rate (TP/(TP + FN)) is the proportion of all the positives predicted correctly. F-measure or F1 score is considered as an average of precision and recall and can be calculated as ((2 x Precision x Recall)/(Precision + Recall). MCC is a correlation coefficient between the experimental and the predicted classifications and is computed to introduce a balance in the predictions made by the classifiers in case of classes of varying sizes.

### Screening of anti-Alzheimer drugs against the novel and known Alz-associated genes

A list of 45 already existing approved and investigational drugs specific to Alzheimers was retrieved from the DrugBank [48] database and chemical structures of a total of 37 drugs were obtained from the PubChem compound database. DrugBank is a freely available online database that houses information on a broad category of drugs and drug targets. Using the Glide [49, 50] docking module available from Schrodinger [51], we carried out extra-precision (XP) docking studies using the predicted and already known Alz-associated genes as drug targets into which 37 Alzheimer specific drugs were docked. A thorough Protein Data Bank (PDB) [52] search was performed to download the three-dimensional crystal structures of the predicted novel targets along with the structures for the three well-established Alzheimer genes, APOE, APP and PSEN1. The PDB structures were preprocessed using Schrodinger’s Protein Preparation Wizard [51, 53] prior to which the water molecules and heteroatoms were removed from the structures using Accelrys ViewerLite (Accelrys, Inc., San Diego, CA, USA). The protein preprocessing steps included adjustment of bond orders, cofactors and metal ions, assignment of correct formal charges, hydrogen bonds addition and protein termini capping followed by a restrained energy minimization of the protein. A receptor grid was generated centered on the active site residues provided by the user using the Receptor Grid Generation panel of Schrodinger [54, 55]. The 37 Alzheimer specific drugs were used as ligands and were prepared using the LigPrep [56] program available from Schrodinger. The other parameters were kept as default for the molecular docking studies. The best docked pose of each ligand was selected for each protein to be used for MD simulation study further.

### Understanding protein-ligand complex behavior through molecular dynamics simulations

Post molecular docking, the docked protein-ligand complexes for the novel targets were subjected to MD simulation studies to evaluate the stability of the ligand and protein in the presence of salt and the solvent [57]. The MD simulation studies were performed using Desmond Molecular Dynamics [58] platform. The docked protein-ligand complexes were first refined using Protein Preparation Wizard followed by generation of a solvated system that included the protein-ligand complex as solute and the water molecules as solvent, using simple point charge as water model. The box shape was kept as Orthorhombic, the buffer region containing the solvent molecules was kept at 10 Å distance from the protein atoms and the volume of the generated solvent was minimized to reduce the duration of the simulation process. Further, the protein-ligand complexes were subjected to 2000 steps of energy minimization using Steepest Descent (SD) algorithm until a gradient threshold of 25 kcal/mol/Å, and Optimized Potentials for Liquid Simulations (OPLS) all-atom force field 2005 [59, 60] with a constant temperature 300 K and 1 bar pressure. A 25 ns MD simulation was then performed using Berendsen algorithm and Isothermal–isobaric (NPT) ensemble at constant temperature (300 K) and pressure conditions (1 atm). Post MD simulation, the protein-ligand complexes were visualized using Schrodinger’s maestro and root mean square deviation (RMSD) analysis was carried out for all the simulated complexes.

### MM/GBSA method to calculate binding free energies

To calculate the relative binding affinities of the ligands with the targets, MM/GBSA calculations were carried out using Schrodinger [61]. MM/GBSA is a widely used computationally efficient method to compute the binding free energy of a set of ligands to a protein and is based upon

$$ \Delta \mathrm{G}\ \left(\mathrm{binding}\right) = \mathrm{Energy}\ \mathrm{complex}\ \left(\mathrm{minimized}\right)\ \hbox{-}\ \left(\mathrm{Energy}\ \mathrm{ligand}\ \left(\mathrm{minimized}\right) + \mathrm{Energy}\ \mathrm{receptor}\ \left(\mathrm{minimized}\right)\right) $$

The protein-ligand complexes obtained after MD simulation analysis were used as input for MM/GBSA calculation.

## Results and Discussion

In the present study we have tried to identify potential Alz genes based on the extraction of their network, sequences and functional properties using machine learning approaches. We have carried out feature selection using seven different feature selection techniques along with PCA to extract significant features and used 11 machine learning classifiers to predict candidate Alz genes. To do so, we have obtained a list of known Alz-associated and NonAlz genes from the Entrez Gene database, which made the positive and negative dataset respectively. We also performed a series of docking studies followed by MD and MM/GBSA calculation and screened the already existing approved and investigational anti-Alzheimer drugs to identify drugs against novel candidate genes.

### Analysis of various biological features for Alz-associated and NonAlz genes

#### Network features

A total of nine topological properties were calculated for each gene in the PPI datasets and a comparison of the properties between Alz and NonAlz genes was performed. Our results showed that the mean value of the degree for the Alz genes was considerably larger than the NonAlz genes which confirmed a previous finding that disease genes have higher degree value (*P*-value = 0.00002) [62, 63]. The median neighborhood connectivity value was much higher for the non-disease genes (108.7) as compared to the disease genes (88.4) owing to the large number of non-disease genes. However, calculating the average of similar number of samples of disease and non-disease genes further indicates the greater likelihood of neighbors of a disease gene being the other disease genes [62, 64]. We also found that disease proteins have more significant interactions with other proteins in the network as indicated by a very high mean of radiality for disease genes with a significant *P*-value of 0.00006. The mean values of the shortest path to Alz genes, clustering coefficient, topological coefficient, eccentricity and closeness centrality were similar for the Alz and NonAlz gene datasets. Table 1 shows the medians of the network features along with *p*-values between the Alz gene and NonAlz gene sets.

**Table 1:** Lists the medians of the network features along with *p*-values between the Alz gene and NonAlz gene sets

Network feature	Alz genes	NonAlz genes	*p*-value
Average shortest path length	4.10	4.19	6.79E-05
Closeness centrality	0.24	0.23	1.88E-04
Clustering coefficient	0.03	0.06	1.91E-08
Degree	19	13	2.29E-05
Eccentricity	18	18	0
Neighborhood connectivity	88.4	108.7	1.18E-05
Topological coefficient	0.07	0.08	9.17E-02
Radiality	0.87	0.86	6.37E-05

#### Sequence features

A statistical comparison between the sequence properties for Alz and NonAlz genes was also performed which provided us interesting results. The mean value of charge on amino acids was much higher for non-disease genes suggesting that disease genes targets majorly included more hydrophobic and less polar amino acids (*P*-value = 1.64E-07). The more number of arginine residues in non-disease genes also explains the same. The average number of residues for disease genes (491) and non-disease genes (443) confirmed that disease drug targets are longer than non-disease drug targets. The mean value of molecular weight of the Alz proteins (54349.54 Da), was also higher than NonAlz proteins (49547.60 Da) with a significant *P*-value of 0.01. The mean value of isoelectric point was lower for Alz proteins as compared to NonAlz proteins with the values being 6.60 and 7.22 respectively and *P*-value of 3.06E-08 which was due to more number of positively charged amino acids. Table 2 lists the medians of the sequence features and the *p*-values between the Alz proteins and NonAlz proteins sets.

**Table 2:** Shows the medians of the sequence features and the *p*-values between the Alz proteins and NonAlz proteins sets

Sequence feature	Alz genes	NonAlz genes	*p*-value
Molecular weight	54349.54	49547.60	1.61E-02
Residues	491	443	1.49E-02
Average residue weight	111.83	111.90	3.09E-01
Charge	1	4	1.64E-07
Isoelectric Point	6.60	7.22	3.06E-08
A280 Molar Extinction Coefficients	50880	44380	7.66E-05
A = Ala	6.81	6.85	7.98E-01
F = Phe	3.77	3.56	1.48E-02
L = Leu	9.38	9.81	2.01E-02
N = Asn	3.78	3.46	1.22E-04
P = Pro	5.33	5.52	5.42E-02
R = Arg	5.09	5.55	4.89E-06
S = Ser	7.53	7.59	2.97E-01
T = Thr	5.31	5.04	6.63E-04
Aliphatic	27.7	27.6	6.34E-01
Polar	47.0	47.2	5.28E-01
Non-polar	52.9	52.7	5.28E-01
Small	50	49.3	3.80E-02
Basic	13.46	13.99	1.82E-04
Aromatic	10.63	10.15	4.97E-02
Acidic	11.94	11.73	3.64E-02

#### Functional features

We retrieved GO terms and Swiss-Prot functional annotation terms using Gene Functional Classification module implemented in the DAVID tool and obtained GO terms distributed into three categories, i.e. molecular function, cellular component and biological process. Among the biological process, the terms strongly associated with disease/Alz genes comprised cell death and apoptosis and their regulation (positive and negative) related terms, response to endogenous stimulus and organic substance, phosphorylation and its regulation, and metabolic processes and their regulation which clearly states that the AD related genes are largely involved in neuronal death [65]. The NonAlz genes terms included transcription and regulation of transcription. The terms favored for cellular component, in case of Alz genes, included plasma membrane part, cell fraction, membrane fraction and insoluble fraction, enzyme binding, vesicle, cytoplasmic, membrane-bounded and cytoplasmic membrane-bounded vesicle, cell projection, and neuron projection. In case of NonAlz genes, the cellular component terms involved organelle membrane, organelle envelope and organelle lumen, nuclear lumen, and cytosolic part. This indicated that the disease drug targets are not localized within the organelles as is reflected for non-disease targets, and are extracellular [66]. For the molecular function, terms associated with Alz genes are identical protein binding and enzyme binding which suggests that disease drug targets are associated with binding and are mostly enzymes [67]. The favorable terms for NonAlz genes included nucleotide binding and purine nucleotide binding.

### Extraction of features contributing to Alz genes classification

In order to detect the features that contribute significantly towards distinguishing between disease genes and non-disease genes, we used seven feature selection techniques on an initial set of 92 features. We identified a final subset of 33 features which were selected by five out of seven selection algorithms and had loadings value >0.1 in PCA, indicating their association with AD (Table 3). The feature selection was performed on the combined dataset of Alz- and NonAlz-associated genes and the complete lists of features obtained after each selection technique are available as Additional file 3: Table S2. Post feature selection, the Alz- and NonAlz-associated genes dataset was divided into a training set containing 11021 genes and a testing set of 2755 genes which were used as the input to the classifier model systems which could predict the potential disease genes.

**Table 3:** Selected features obtained after applying feature selection techniques

Features category		
Network features	Sequence features	Functional features
Clustering Coefficient	Charge	GO:0006916 ~ anti-apoptosis
Degree	Isoelectric Point	GO:0010942 ~ positive regulation of cell death
Average Shortest Path Length	R = Arg	GO:0043068 ~ positive regulation of programmed cell death
Closeness Centrality	Acidic	GO:0043066 ~ negative regulation of apoptosis
Neighborhood Connectivity		GO:0009725 ~ response to hormone stimulus
		GO:0009719 ~ response to endogenous stimulus
		GO:0043005 ~ neuron projection
		GO:0010941 ~ regulation of cell death
		GO:0010033 ~ response to organic substance
		GO:0032268 ~ regulation of cellular protein metabolic process
		GO:0019899 ~ enzyme binding
		Mutagenesis site
		GO:0044093 ~ positive regulation of molecular function
		GO:0008219 ~ cell death
		Transmembrane protein
		Lipoprotein
		Active site: Proton acceptor
		GO:0016023 ~ cytoplasmic membrane-bounded vesicle
		GO:0042802 ~ identical protein binding
		GO:0031982 ~ vesicle
		Disease mutation
		GO:0042127 ~ regulation of cell proliferation
		GO:0000267 ~ cell fraction
		GO:0005624 ~ membrane fraction

### Performance of the classifiers generated to predict Alz-associated genes

Various machine learning algorithms, which have been widely used for classification purposes, were used to build the model systems using training set which could classify the disease genes and non-disease genes from the test set using the final set of contributing features. Using 11 machine learning algorithms, a total of 22 model systems were generated, 11 models using standard classifiers and 11 using cost-sensitive classifiers employing confusion matrix, and their performances were evaluated using various statistical indices. The 11 cost-sensitive classifier models outperformed the standard classifier models as can be seen in Additional file 4: Table S3. Tables 4 and 5 list the number of prediction by the cost sensitive classifier algorithms and results of the indices used to measure the performance of the classifiers, respectively. All the classifiers performed well having an accuracy of around 75 % and false positive rate of around 20 % during 10-fold cross-validation. Another popular measure, F-Measure, was also calculated which came out to be highest for NB (0.15) classifier followed by LR (0.14) and SVM (0.14) classifiers. The SVM classifier had the highest recall value of 78.8 % followed by the NB and LR classifiers for which it was 71.8 % and 69 % respectively, as compared to the other classifiers. The three classifiers, NB, LR and SVM also had good MCC values, which were 0.20, 0.19 and 0.20 correspondingly. The results presented in the current study can be reproduced easily using the datasets (training set and test set) and the 11 cost-sensitive classifier models generated which are available as Additional file 5.

**Table 4:** Confusion matrix. Predictions by the cost sensitive classifier algorithms on the Entrez Gene dataset

Classifier algorithms	True positives (TP)	True negatives (TN)	False positives (FP)	False negatives (FN)
Bayes Net	47	2110	574	24
Decision Table	19	2032	652	52
DTNB	21	2133	551	50
Functional Tree	46	2004	680	25
J48	44	2117	567	27
Logistic Regression	49	2148	536	22
LWL (J48 + KNN)	48	2111	573	23
Naive Bayes	51	2151	533	20
NB Tree	35	2070	614	36
Random Forest	42	2158	526	29
SVM	56	2058	626	15

**Table 5:** Performance of the cost sensitive classifier algorithms on the Entrez gene dataset

Classifier algorithms	TP rate/Recall	FP rate	Accuracy	Precision	F-measure	MCC
Bayes Net	0.662	0.214	0.782	0.076	0.136	0.169
Decision Table	0.268	0.243	0.744	0.028	0.051	0.009
DTNB	0.296	0.205	0.781	0.037	0.065	0.035
Functional Tree	0.648	0.253	0.744	0.063	0.115	0.141
J48	0.620	0.211	0.784	0.072	0.129	0.155
Logistic Regression	**0.690**	0.20	**0.797**	**0.084**	**0.149**	**0.190**
LWL (J48 + KNN)	0.676	0.213	0.783	0.077	0.139	0.175
Naive Bayes	0.718	0.199	**0.799**	**0.087**	**0.156**	**0.201**
NB Tree	0.493	0.229	0.764	0.054	0.097	0.098
Random Forest	0.592	0.196	**0.798**	0.074	0.131	0.154
SVM	**0.788**	0.233	0.767	**0.082**	**0.148**	**0.203**

The genes predicted to be probable Alz genes by all the 11 cost-sensitive model systems were considered for further analysis in the study which resulted in a total of 13 genes (Table 6). The 13 predicted probable Alz genes include Cadherin 1: type 1 (CDH1), Caspase recruitment domain family: member 8 (CARD8), Coagulation factor VII (F7), Intersectin 1 (ITSN1), Janus kinase 2 (JAK2), Nuclear factor of kappa light polypeptide gene enhancer in B-cells inhibitor: alpha (NFKBIA), Phospholipase C: gamma 2 (phosphatidylinositol-specific) (PLCG2), Ras homolog family member A (RHOA), Receptor-interacting serine-threonine kinase 3 (RIPK3), Retinoblastoma 1 (Rb1), Signal transducer and activator of transcription 5A (STAT5A), Tubulin: beta class I (TUBB) and Vinculin (VCL). The network topological features, sequence features and functional properties for the 13 genes have been provided as Additional file 6: Table S4. We could not find experimental evidences in support of association between all predicted novel Alz genes and AD, such genes include F7 and VCL.

**Table 6:** List of the candidate genes predicted to be Alzheimer’s associated by all the classifier algorithms

Entrez ID	Official gene symbol	Official gene name
999	CDH1	Cadherin 1, type 1
22900	CARD8	Caspase recruitment domain family, member 8
2155	F7	Coagulation factor VII (serum prothrombin conversion accelerator)
6453	ITSN1	Intersectin 1 (SH3 domain protein)
3717	JAK2	Janus kinase 2
4792	NFKBIA	Nuclear factor of kappa light polypeptide gene enhancer in B-cells inhibitor, alpha
5336	PLCG2	Phospholipase C, gamma 2 (phosphatidylinositol-specific)
5925	RB1	Retinoblastoma 1
387	RHOA	Ras homolog family member A
11035	RIPK3	Receptor-interacting serine-threonine kinase 3
6776	STAT5A	Signal transducer and activator of transcription 5A
203068	TUBB	Tubulin, beta class I
7414	VCL	Vinculin

### Understanding association between novel Alz genes and Alzheimers

We looked for experimental evidences to support the role of novel Alz genes in AD and found that various studies have reported that the cadherins play an important role in regulation of synapses are an important players in production of Aβ which is the major hallmark in AD [68]. The localization of Presinilin-1 (PS1) at synaptic sites and formation of complexes with Cadherin/catenin regulating their functions and the further dissociation of the complex by a PS1/γ-secretase activity [69, 70] results in the trafficking of N- and E-cadherin in the cytoplasm which encourages the dimerization of amyloid precursor protein (APP) resulting in increased extracellular release of Aβ [71].

Caspases, cysteine aspartyl-specific proteases, have been proposed as potential therapeutic targets for the treatment of AD brain disorder and a lot of inhibitors have been investigated [72, 73]. Aβ has been suggested to activate caspase-8 and caspase-3 which are the key players in neuronal apoptosis and thus may be involved in neurodegenerative disorders [74].

There have been growing evidences which indicate that the JAK2/STAT3 intracellular signaling pathway has significant involvement in memory impairment in AD and have explored the effect of Aβ on JAK2/STAT3 pathway [75]. Elevated levels of Aβ lead to the inactivation of JAK2/STAT3 pathway in the hippocampal neurons causes’ memory loss and further AD which can be reversed by a recently proposed novel 24-amino acid peptide, Humanin (HN), and its derivative, colivelin (CLN). These studies clearly indicate the role of JAK2/STAT3 signaling axis in AD and thus JAK2, STAT3 and STAT5 may be considered as novel targets in AD therapy which could be studied in-length to gain insights into mechanism of JAK2/STAT3 activation [76–79].

Inflammatory process has been accounted for the Alzheimer’s disorder since long back and NF-kB has been considered as an important regulator of inflammation. Activation of NF-kB is involved in many other neurodegenerative disorders say Huntington disease, Parkinson disease along with the AD where Aβ is accounted for NF-kB upregulation [80]. Acetylcysteine, a FDA-approved drug, is already in use for the treatment of AD and it has been shown to suppress NF-kB activation and thus making NF-kB as principal target of Acetylcysteine [81].

The overexpression of PLCG2 on phosphatidylinositol 4, 5-bisphosphate (PIP_2_) stimulates generation of inositol 1, 4, 5-trisphosphate (IP) further resulting in enhanced Ca^2+^ concentration [82]. Another study also examined and found increased levels of PLCG2 in brains of AD patients which puts forwards PLCG2 as an important target in pathophysiology of AD [83].

Numerous studies have suggested that the Down syndrome (DS) patients develop multiple conditions, one among which is AD and that the genes overexpressed in case of DS can be considered as novel therapeutic targets against AD [84]. ITSN1 is one such gene overexpression of which prevents clatherin-mediated endocytosis which is an essential process for recycling of synaptic vessels [85].

RhoA, a small GTPase protein known to regulate synaptic strength and plasticity, has also been pointed out as a key therapeutic target in AD pathogenesis through RhoA GTPase/ROCK (Rho-associated protein kinase) pathway [86]. RhoA-ROCK pathway has been implicated in Aβ production and inhibition of neurite outgrowth by Aβ thus suggesting Rho-ROCK inhibition helpful for AD patients [86, 87].

Necroptosis is a significant cell death mechanism which is involved in many neurodegenerative disorders including AD [88]. RIPK3 is a member of family of serine-threonine protein kinases and has a critical role in NF-kB activation and inducing apoptosis [89].

A wide range of studies have reported that increased levels of a specific miRNA, miR-26b, may play a vital role in pathogenesis of AD suggesting a connection amid cell cycle entry and tau aggregation [90, 91]. The miR26-b also activates cyclin-dependent kinase-5 (Cdk5), dysregulation of which has been implicated in AD pathogenesis [92].

Rb1 is a tumor-suppressor protein and major target of miR-26B, which controls cell growth by inhibiting transcription factor, E2F required for further transcription of genes. Cdk5 causes hyper-phosphorylation of Rb1 upon which it is unable to bind to E2F and consequently E2F transcriptional targets, that include genes for cell cycle, are highly expressed [93]. Thus it becomes clear that alteration in Rb1/E2F signaling pathway and therefore overexpression of Rb1 and E2F target genes leads to abnormal CCE and enhanced tau-phosphorylation causing apoptotic death of neurons and AD.

TUBB protein is a principal constituent of microtubules which are formed by polymerization of dimers of α-tubulin and β-tubulin for which α- and β-tubulin bind to Guanosine-5′-triphosphate (GTP). It has been reported that higher levels of β-tubulin can be associated with aberrant hyper-phosphorylated tau aggregates which play a major role in etiology of AD [94].

### Exploring interactions between known Alz genes and the predicted ones

**Fig. 1:**
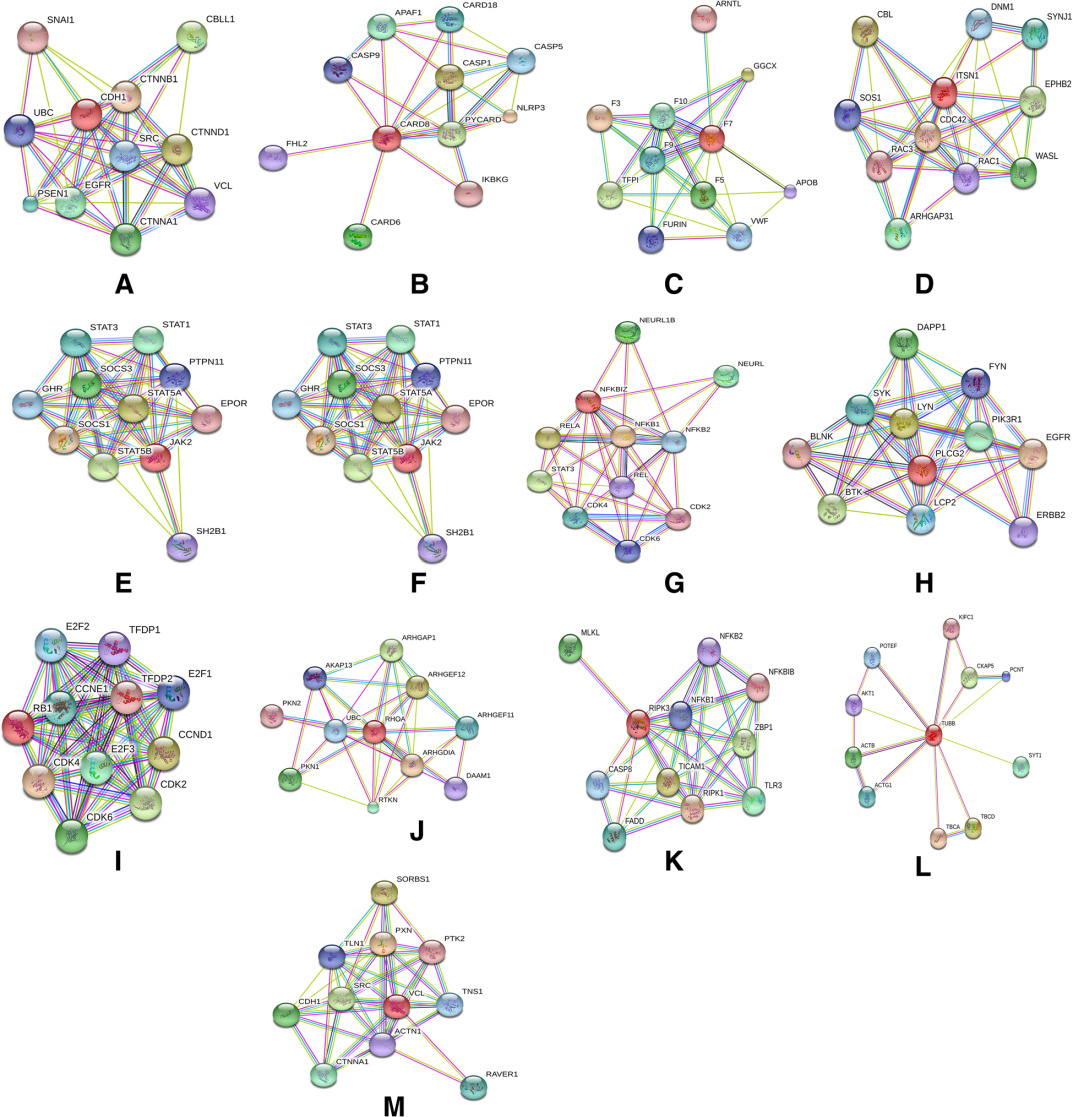
Depicts the interaction networks between the already established Alzheimer genes and the 13 novel genes predicted in the present study. **a** CDH1 (**b**) CARD8 (**c**) F7 (**d**) ITSN1 (**e**) JAK2 (**f**) STAT5 (**g**) NFKBIA (**h**) PLCG2 (**i**) Rb1 (**j**) RHOA (**k**) RIPK3 (**l**) TUBB (**m**) VCL

Using STRING database we generated interaction networks and explored the associations between the already known Alz genes and the 13 novel Alz genes identified in the present study. We found the interactions for all the predicted genes except CDH1, CARD8, RHOA and VCL. F7 was found to be interacting with apolipoprotein B (APOB) which was present in high concentrations in AD patients [95]. ITSN1 interacted with dynamin 1 (DNM1) which is essential for information processing but is depleted by Abeta in case of Alzheimer’s [96]. JAK2 interacted with protein tyrosine phosphate (PTPN), the levels of which were found to be increased in AD [97] and erythropoietin receptor (EpoR), upregulation of which was observed in case of sporadic AD [98]. NFKBIA interacted with CDK which has been discussed earlier and REL which is a subunit of NF-kB and controls the expression of APP [99]. PLCG2 interacted with two Alzheimer associated genes, fibroblast yes related novel (FYN) gene which codes FYN kinase and is activated by abeta and is elevated in AD [100] and ErbB also known as epidermal growth receptor factor. Insufficient ErbB signaling has been associated with the development of Alzheimers [101]. The interaction of Rb1 with E2F1 and CDK has been discussed earlier in the present study. STAT5 interacted with EpoR and the upregulation of EpoR has a significant role in the pathogenesis of Alzheimer’s [98]. TUBB showed interaction with Akt which was overexpressed in case of AD [102]. Figure 1 depicts the interaction networks between the already established Alzheimer genes and the 13 novel genes predicted in the present study.

### Prioritization of anti-Alzheimer drugs against the novel and known Alz targets

In order to identify drugs against the predicted novel Alz-associated targets, we employed molecular docking approach and screened a total of 37 already known Alz-specific drugs against the novel target genes. Among the 13 Alz-associated genes identified, the crystal structures were available only for seven and the same were downloaded from PDB. A list of the existing approved and investigational Alz-specific drugs (Additional file 1: Table S1) and the information on PDB structures (Additional file 3: Table S2) has been provided in Additional file 7. We observed that an investigational drug, AL108 (PubChem CID: 9832404) showed high binding affinity (glide score > –6.5 kcal/mol) towards all the targets excluding NFKBIA for which another investigational drug, PPI-1019 (PubChem CID: 44147342) showed significantly greater binding affinity (glide score, –6.41 kcal/mol). AL108 exhibited highest binding affinity for JAK2 with a binding score of –10.87 kcal/mol followed by RIPK3 (–8.99 kcal/mol), RhoA (–8.68 kcal/mol), Cadherin (–8.34 kcal/mol), Rb1 (–7.07 kcal/mol) and lowest for Card8 (–6.90 kcal/mol). Other than for NFKBIA, PPI-1019 also had strong binding affinity for all the other targets. Additional file 7 (Additional file 4: Table S3) provides detailed docking results for all the Alz-associated drug targets. Table 7 provides the glide docking scores and MMGBSA energy values for the top scoring compounds against seven novel candidate Alz-associated genes. Additional file 8: Figure S2 and Additional file 9: Figure S3 depict the interaction patterns of the ligands within the active site of the novel candidate Alzheimer protein targets. Additionally, we mapped all the 13 candidate Alz-associated genes to the already known anti-Alzheimer drug targets and identified the NFKBIA gene to be targeted by the approved drug, Acetylcysteine. We also performed molecular docking studies on the already known Alz-genes, APOE, APP and PSEN1 and it was observed that AL108, an investigational drug, shown strong binding affinity towards APOE (–5.30 kcal/mol) and PSEN1 (-6.95 kcal/mol). APP showed strong interaction with another known anti-Alzheimer drug, Leuprolide (PubChem CID: 657181) with glide score of –7.67 kcal/mol followed by AL108 having docking score, –6.97 kcal/mol.

**Table 7:** Docking scores and MMGBSA energy values for the top scoring compounds against seven novel candidate Alz-associated genes

Candidate Alzheimer target	Docked compound	Glide score (kcal/mol)	ΔG (binding) (kcal/mol)
Cadherin 1	AL-108	–8.34	–58.92
Caspase recruitment domain family, member 8	AL-108	–6.90	–36.50
Janus kinase 2	AL-108	–10.87	–74.34
Nuclear factor of kappa light polypeptide gene enhancer in B-cells inhibitor, alpha	PPI-1019	–6.41	–13.66
Retinoblastoma 1	AL-108	–7.07	–12.09
Ras homolog family member A	AL-108	–8.68	–49.84
Receptor-interacting serine-threonine kinase 3	AL-108	–8.99	–77.07

### Molecular dynamics simulations analysis

**Fig. 2:**
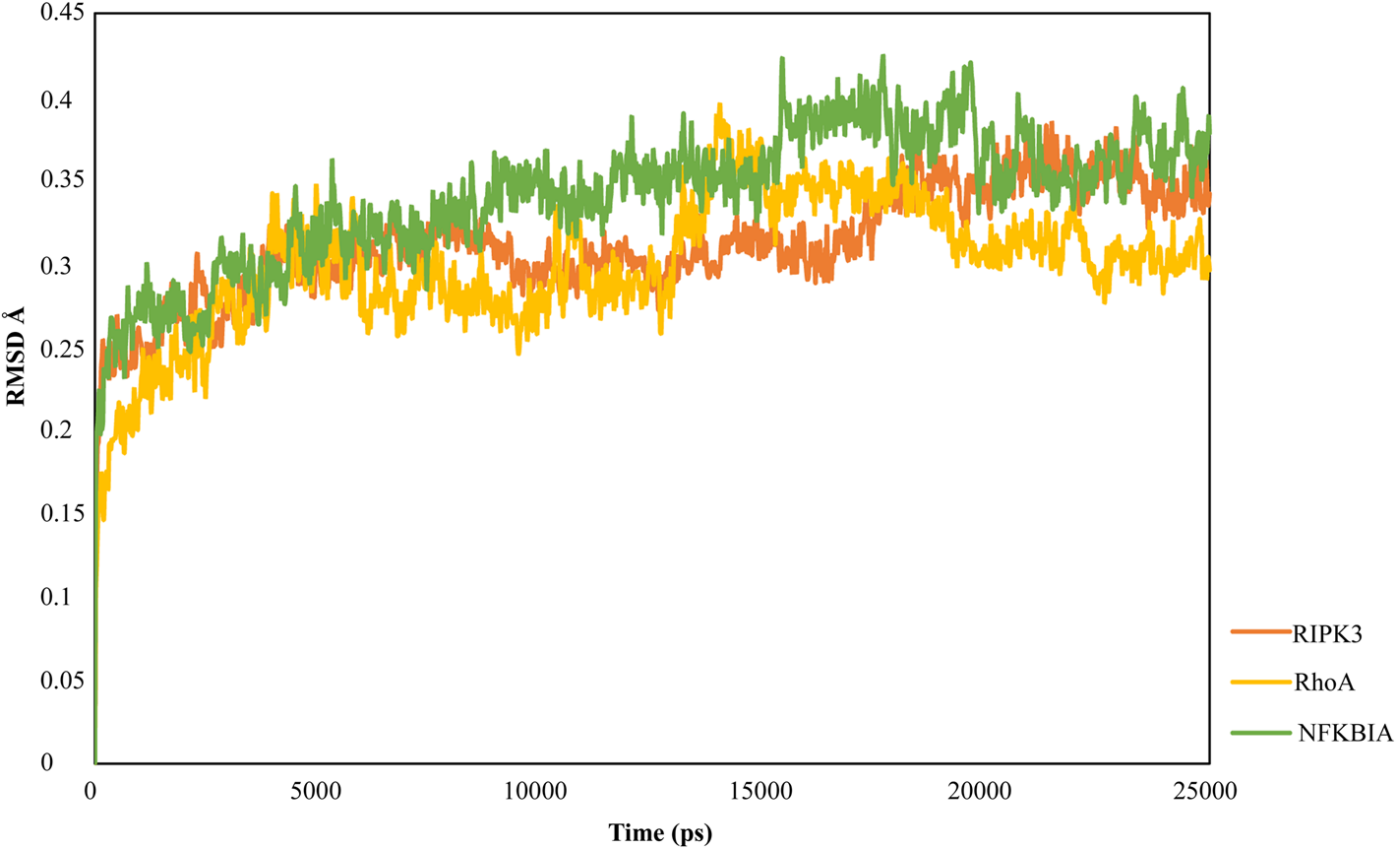
Shows the RMSD plot of RIPK3, RhoA and NFKBIA

**Fig. 3:**
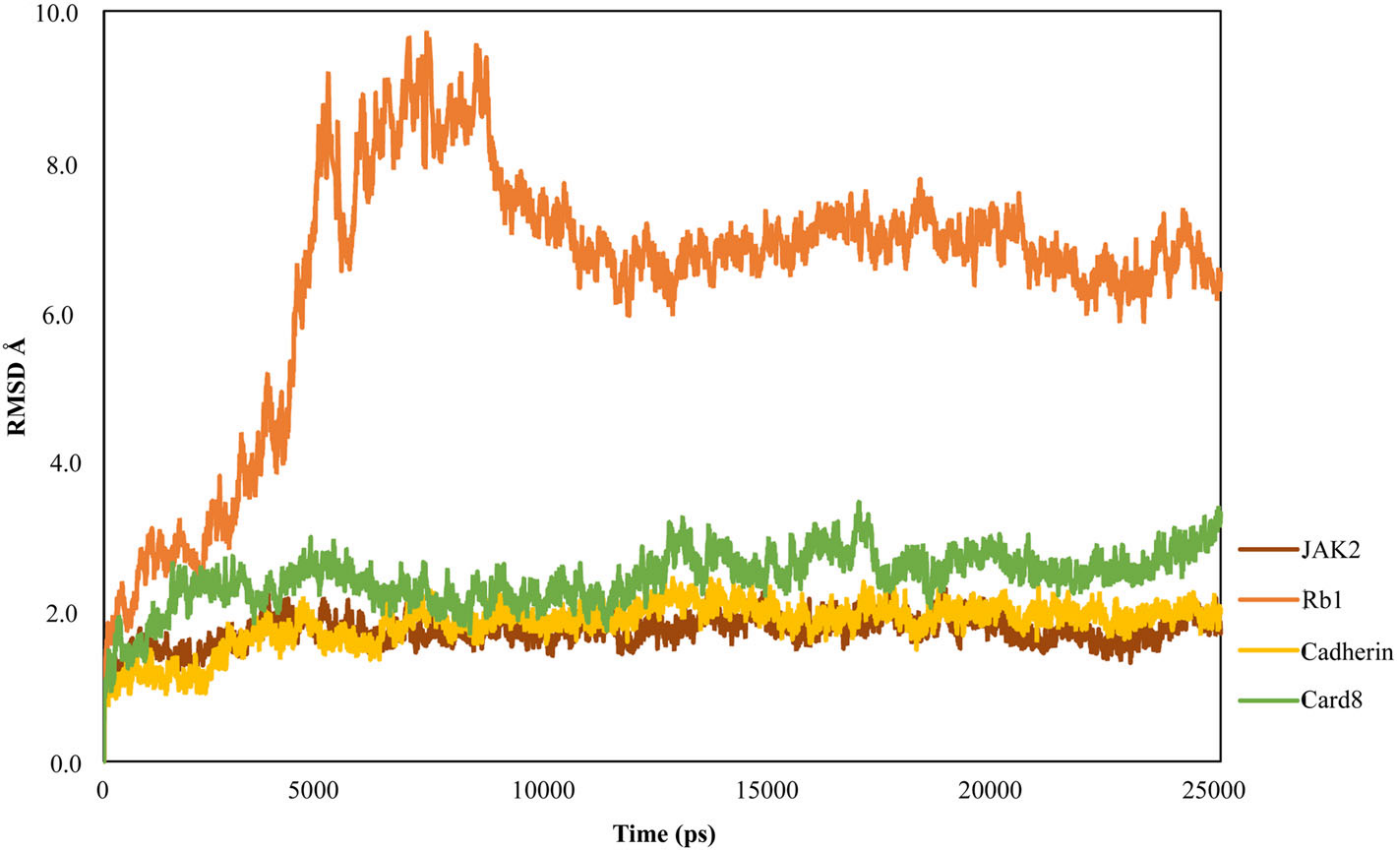
Shows the RMSD plot of JAK2, Rb1, Cadherin and Card8

The seven protein-ligand complexes were subjected to 25 ns long MD simulations to understand the dynamic interaction behavior of the ligand and the active site residues of the target in the presence of the explicit salt and solvent models. We observed that all the complexes had stable root mean square deviation (RMSD) trajectories and no major structural changes were observed. Figures 2 and 3 show the RMSD plot where RMSD values have been plotted against the MD simulation time steps. Stable trajectories for RIPK3, RhoA and NFKBIA were found during 18–25 ns, 19–25 ns and 9–15 ns time durations respectively (Fig. 2). JAK2, Cadherin and Card8 had very good stability throughout the simulation process with RMSD values around 1–2 Å for JAK2 and Cadherin and 2–3 Å for Card8 (Fig. 3). We observed Rb1 to be highly unstable for initial 10 ns after which the complex was found to be stable till 25 ns with RMSD value 6–7 Å (Fig. 3). The post-MD simulation interaction patters of the ligands with the residues of the binding sites of proteins have been shown in Additional file 10: Figure S4 and Additional file 11: Figure S5.

### Binding free energies calculations

The MD simulated protein-ligand complexes were used to calculate the binding free energies and we found that the binding of AL-108 was thermodynamically favorable for all the drug targets. The Rb1-AL108 complex had the highest free energy value –13.66 kcal/mol followed by NFKBIA-AL108 with binding energy –12.09 kcal/mol. Table 7 provides the computed binding free energies for AL-108 and the novel candidate drug target complexes.

### Using the classifiers on human genome epidemiology network (HuGENet) dataset

The 11 machine learning classifiers generated were applied to identify the Alz genes from the HuGENet repository. A total of 1686 Alz-associated genes were obtained among which 1304 genes were found to be the part of the training and testing set used for model systems generation and validation respectively. The resulting 382 genes, which were not the part of disease and non-disease gene lists, were used to calculate the network, sequence and functional features. Further, 39 genes were given as input to the 11 trained classifiers and a majority of the models gave around 60 % correct predictions among which the SVM classifier was 97.4 % accurate. Additional file 12: Table S6 provides the information on the predictions made by the 11 classifiers on 39 HuGENet genes.

## Conclusion

Alzheimer’s, a highly complex neurological disorder, has become the cause of serious global concern owing to the rapidly increasing number of cases and the socioeconomic burden associated with it. The pathogenesis of the disease is still not clear and thus no effective treatments to cure the disease exist so far. However, a plethora of studies have stated genetic factors as the major cause of the disease in light of which identification of novel Alz genes will be of great significance to understand disease etiology and in order to develop effective therapeutics. The computational predictive models generated in the present study successfully identified 13 novel candidate genes that could have a potential role in AD pathology. We incorporated various properties of the genes, network properties from the signaling pathways, sequence properties from the corresponding protein sequences and functional annotations and employed eleven machine learning algorithms to train the model systems. Additionally, we used a molecular docking approach followed by MD simulations and performed a screening of already available anti-Alzheimer drugs against the novel predicted Alz drug targets. Finally, MMMGBSA calculations were performed and the obtained binding free energy values showed that AL-108, an investigational AD-specific drug, had strong binding affinity majorly for all the novel drug targets. The investigational drug, AL-108 can be considered as a probable lead compound having inhibitory properties against the novel drug targets identified in the present study. The computational protocol used in the current study can be successfully applied for the prediction of disease associated genes and have insights into the disease mechanisms for the development of better and effective therapeutic agents.

## Additional files

Additional file 1: Table S1. Network, sequence and functional properties computed using Network Analyzer (Cytoscape), Pepstats (Emboss) and DAVID, respectively for Alz and NonAlz genes.
Additional file 2: Figure S1. Shows the percent variation explained by. the first two principal components.
Additional file 3: Table S2. The complete lists of features obtained after each selection technique.
Additional file 4: Table S3. Confusion matrix. Predictions by the individual base classifier algorithms on the Entrez Gene dataset.
Additional file 5: Input datasets (train and test) and the generated models which can be used to reproduce the results presented in the current study.
Additional file 6: Table S4. List of the genes, and their features values, predicted to be Alzheimer’s associated by all the classifier algorithms.
Additional file 7: Table S1. Approved and Investigational anti-Alzheimer drugs downloaded from DrugBank. Table S2: List of the genes, whose crystal structure was available, along with the PDB codes. Table S3: Docking scores of the Approved and Investigational anti-Alzheimer drugs bound to the 7 candidate Alzheimer associated targets. Top scoring drug against each target is in bold highlighted in yellow.
Additional file 8: Figure S2. depicts the interaction patterns of the ligands within the active site of the novel candidate Alzheimer protein targets, Cadherin, CARD8, JAK2 and NFKBIA.
Additional file 9: Figure S3. depicts the interaction patterns of the ligands within the active site of the novel candidate Alzheimer protein targets, Rb1, RhoA and RIPK3.
Additional file 10: Figure S4. Shows the post-MD simulation interaction patters of the ligands with the residues of the binding sites of proteins, Cadherin, CARD8, JAK2 and NFKBIA.
Additional file 11: Figure S5. Shows the post-MD simulation interaction patters of the ligands with the residues of the binding sites of proteins, Rb1, RhoA and RIPK3.
Additional file 12: Table S6. Predictions made by the 11 classifier models on the Alzheimer associated genes downloaded from Human Epidemiology Gene Network (HuGENet). The number in bracket indicates the number of correct predictions made by the classifier.

## Abbreviations

AD: Alzheimer’s disease
ApoE: Apolipoprotein E
APP: Amyloid precursor protein
CARD8: Caspase recruitment domain family: member 8
CDH1: Cadherin 1: type 1
DAVID: Database for annotation visualization and integrated discovery
DTNB: Decision table/Naive Bayes
F7: Coagulation factor VII
FN: False negatives
FP: False positives
GO: Gene ontology
GWAS: Genome-wide association studies
ITSN1: Intersectin 1
JAK2: Janus kinase 2
LR: Logistic regression
MCC: Matthews correlation coefficient
MD: Molecular dynamics
NB: Naive Bayes
NCBI: National Centre for Biotechnology Information
NFKBIA: Nuclear factor of kappa light polypeptide gene enhancer in B-cells inhibitor: alpha
OPID: Online predicted human interaction database
PCA: Principal component analysis
PDB: Protein data bank
PLCG2: Phospholipase C: gamma 2 phosphatidylinositol-specific
PPI: Protein-protein interaction
PSEN1: Presenilin-1
PSEN2: Presenilin-2
Rb1: Retinoblastoma 1
RBF: Radial basis function
RF: Random forest
RHOA: Ras homolog family member A
RIPK3: Receptor-interacting serine-threonine kinase 3
STAT5A: Signal transducer and activator of transcription 5A
SVM: Support vector machine
TN: True negatives
TP: True positives
TUBB: Tubulin: beta class I
UniProtKB: Universal protein resource knowledgebase
VCL: Vinculin
