# Supplementary material for: Integrating network, sequence and functional features using machine learning approaches towards identification of novel Alzheimer genes
Source: BMC Genomics. 2016 Oct 18;17:807. doi: 10.1186/s12864-016-3108-1 (PMC5070370; doi:10.1186/s12864-016-3108-1)
Supplement: Additional file 1: Table S1. — Network, sequence and functional properties computed using Network Analyzer (Cytoscape), Pepstats (Emboss) and DAVID, respectively for Alz and NonAlz genes. [file 12864_2016_3108_MOESM1_ESM.docx]

**Supplementary Table 1**. Network, sequence and functional properties computed using Network Analyzer (Cytoscape), Pepstats (Emboss) and DAVID, respectively for Alz and NonAlz genes.

| **Features Category** | | |
| --- | --- | --- |
| **Network features** | **Sequence features** | **Functional features** |
| Average shortest path length  Betweenness centrality  Closeness centrality  Clustering coefficient  Degree  Eccentricity  Neighborhood connectivity  Topological coefficient  Radiality | Molecular weight  Residues  Average Residue Weight  Charge  Isoelectric Point  A280 Molar Extinction Coefficients  A = Ala  F = Phe  L = Leu  N = Asn  P = Pro  R = Arg  S = Ser  T = Thr  Aliphatic  Polar  Non-polar  Small  Basic  Aromatic  Acidic | GO:0006916~anti-apoptosis  GO:0010942~positive regulation of cell death  GO:0043068~positive regulation of programmed cell death  GO:0043065~positive regulation of apoptosis  GO:0043066~negative regulation of apoptosis  GO:0043069~negative regulation of programmed cell death  GO:0060548~negative regulation of cell death  GO:0009725~response to hormone stimulus  GO:0009719~response to endogenous stimulus  GO:0043005~neuron projection  GO:0010941~regulation of cell death  GO:0043067~regulation of programmed cell death  GO:0042981~regulation of apoptosis  GO:0010033~response to organic substance  GO:0032268~regulation of cellular protein metabolic process  GO:0019220~regulation of phosphate metabolic process  GO:0051174~regulation of phosphorus metabolic process  GO:0019899~enzyme binding  GO:0042325~regulation of phosphorylation  GO:0044093~positive regulation of molecular function  GO:0043085~positive regulation of catalytic activity  GO:0008219~cell death  GO:0016265~death  GO:0012501~programmed cell death  GO:0006915~apoptosis  Transmembrane protein  Lipoprotein  Active site: Proton acceptor  GO:0016023~cytoplasmic membrane-bounded vesicle  GO:0031988~membrane-bounded vesicle  GO:0042802~identical protein binding  GO:0031410~cytoplasmic vesicle  GO:0031982~vesicle  Disease mutation  GO:0042127~regulation of cell proliferation  GO:0006468~protein amino acid phosphorylation  Mutagenesis site  GO:0042995~cell projection  GO:0000267~cell fraction  GO:0005624~membrane fraction  GO:0005626~insoluble fraction  GO:0010604~positive regulation of macromolecule metabolic process  GO:0016310~phosphorylation  GO:0009891~positive regulation of biosynthetic process  GO:0031328~positive regulation of cellular biosynthetic process  GO:0051173~positive regulation of nitrogen compound metabolic process  GO:0010557~positive regulation of macromolecule biosynthetic process  GO:0007242~intracellular signaling cascade  GO:0043933~macromolecular complex subunit organization  GO:0010605~negative regulation of macromolecule metabolic process  GO:0005829~cytosol  GO:0006796~phosphate metabolic process  GO:0006793~phosphorus metabolic process  GO:0005794~Golgi apparatus  GO:0044459~plasma membrane part  ATP-binding  Transferase  GO:0005739~mitochondrion  Nucleotide-binding  Transport  Cytoplasm  Acetylation |
