# Supplementary figures and images for: Integrating network, sequence and functional features using machine learning approaches towards identification of novel Alzheimer genes
Source: BMC Genomics. 2016 Oct 18;17:807. doi: 10.1186/s12864-016-3108-1 (PMC5070370; doi:10.1186/s12864-016-3108-1)

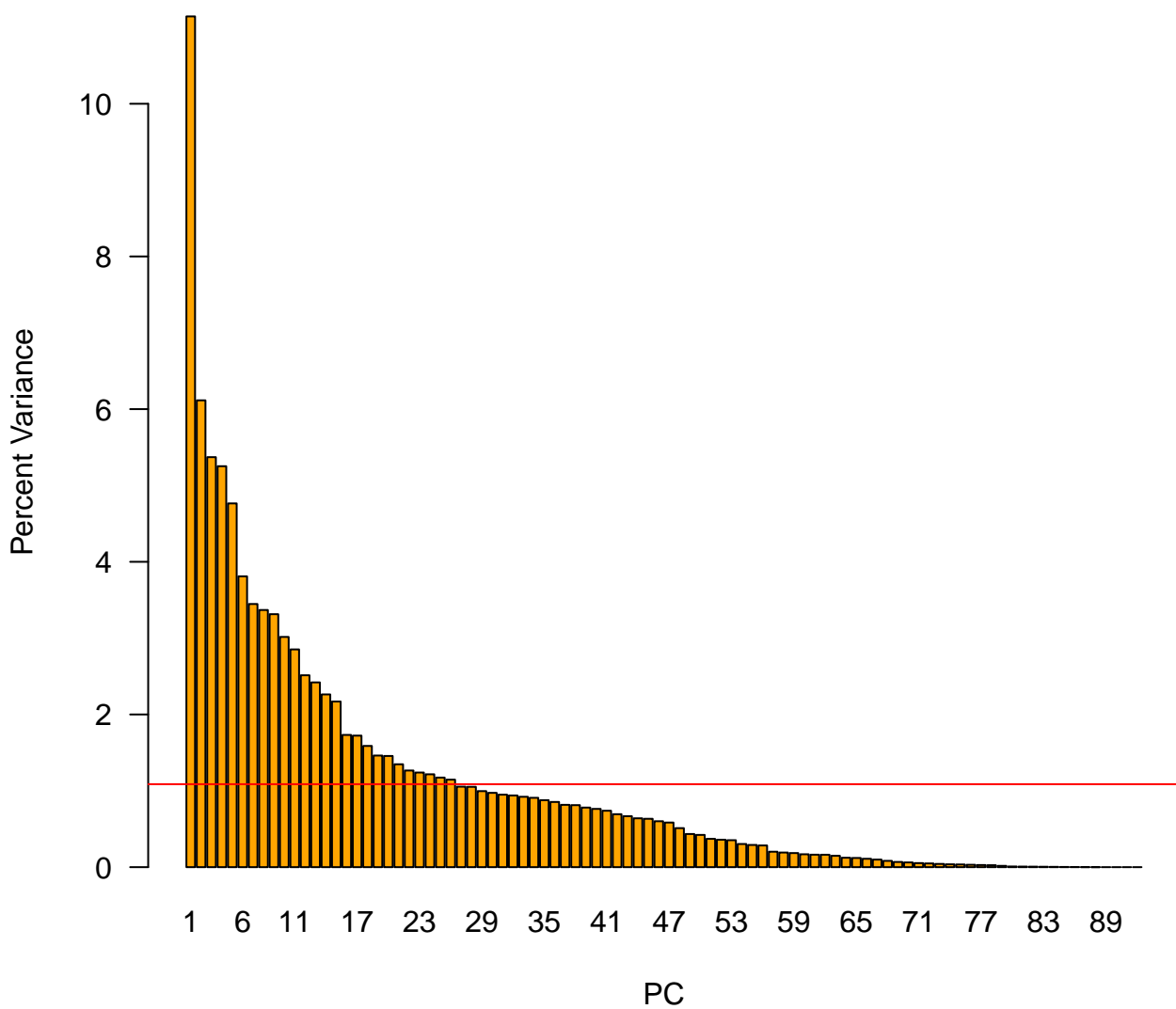

Supplement: Additional file 2: Figure S1. — Shows the percent variation explained by. the first two principal components. [file 12864_2016_3108_MOESM2_ESM.pdf]

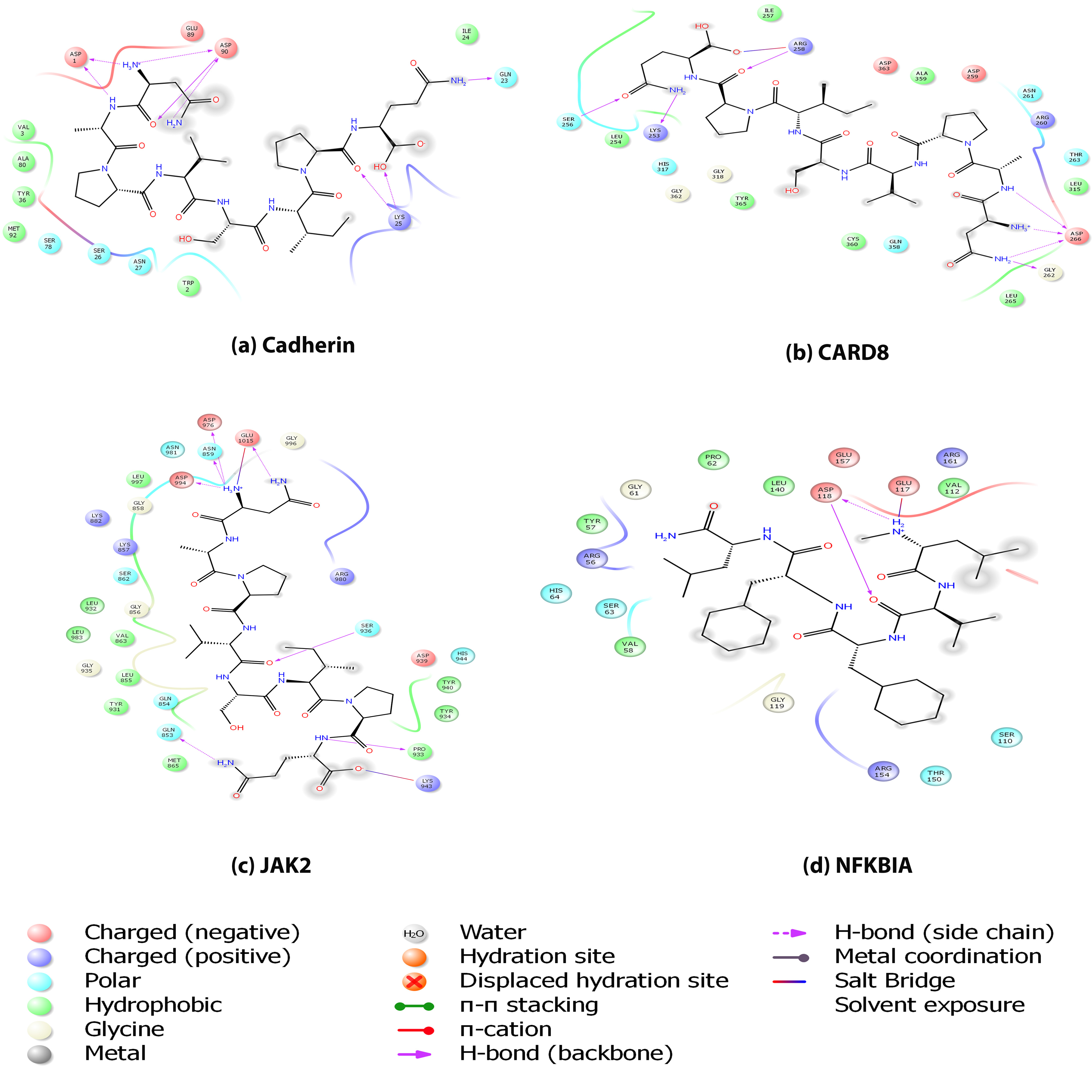

Supplement: Additional file 8: Figure S2. — depicts the interaction patterns of the ligands within the active site of the novel candidate Alzheimer protein targets, Cadherin, CARD8, JAK2 and NFKBIA. [file 12864_2016_3108_MOESM8_ESM.jpg]

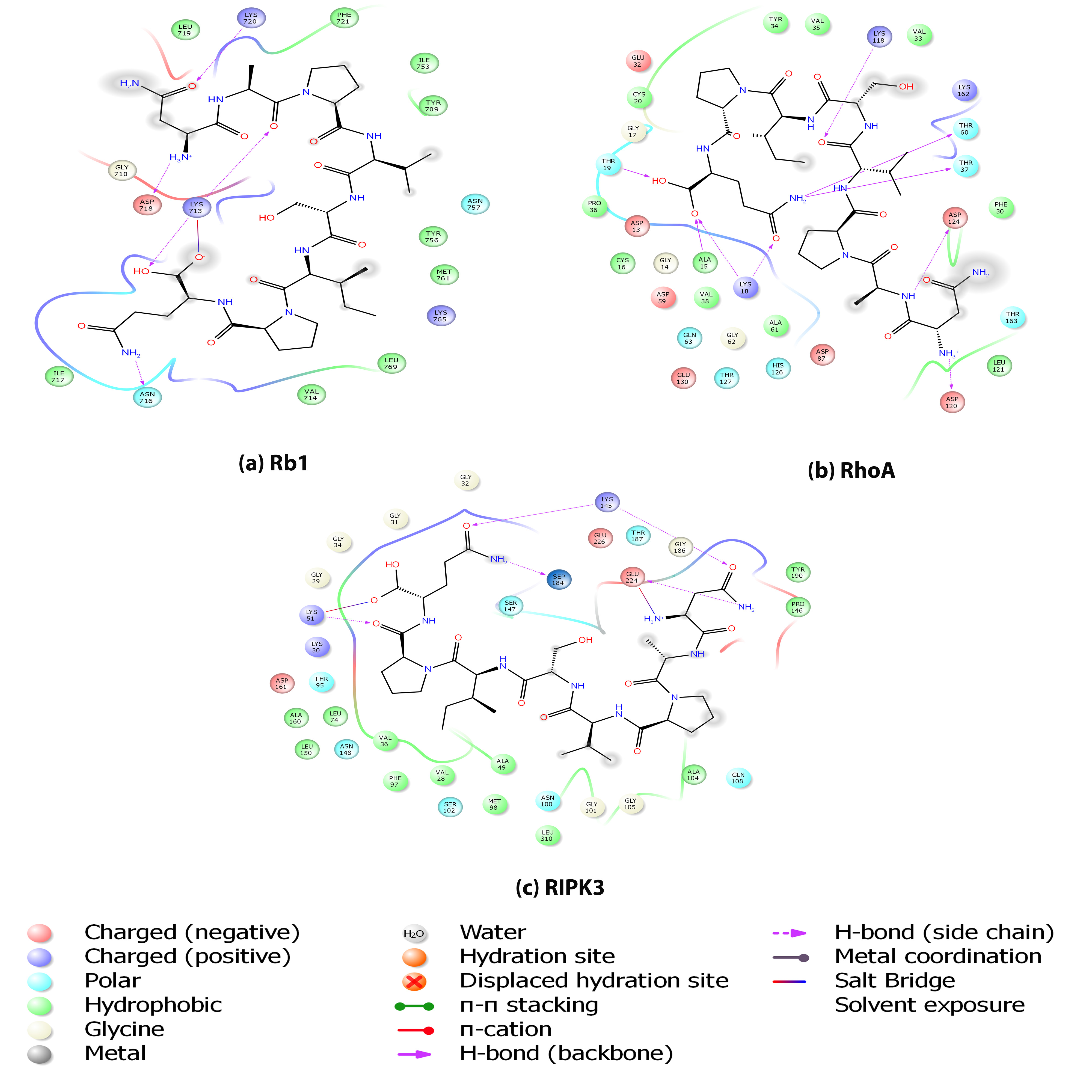

Supplement: Additional file 9: Figure S3. — depicts the interaction patterns of the ligands within the active site of the novel candidate Alzheimer protein targets, Rb1, RhoA and RIPK3. [file 12864_2016_3108_MOESM9_ESM.jpg]

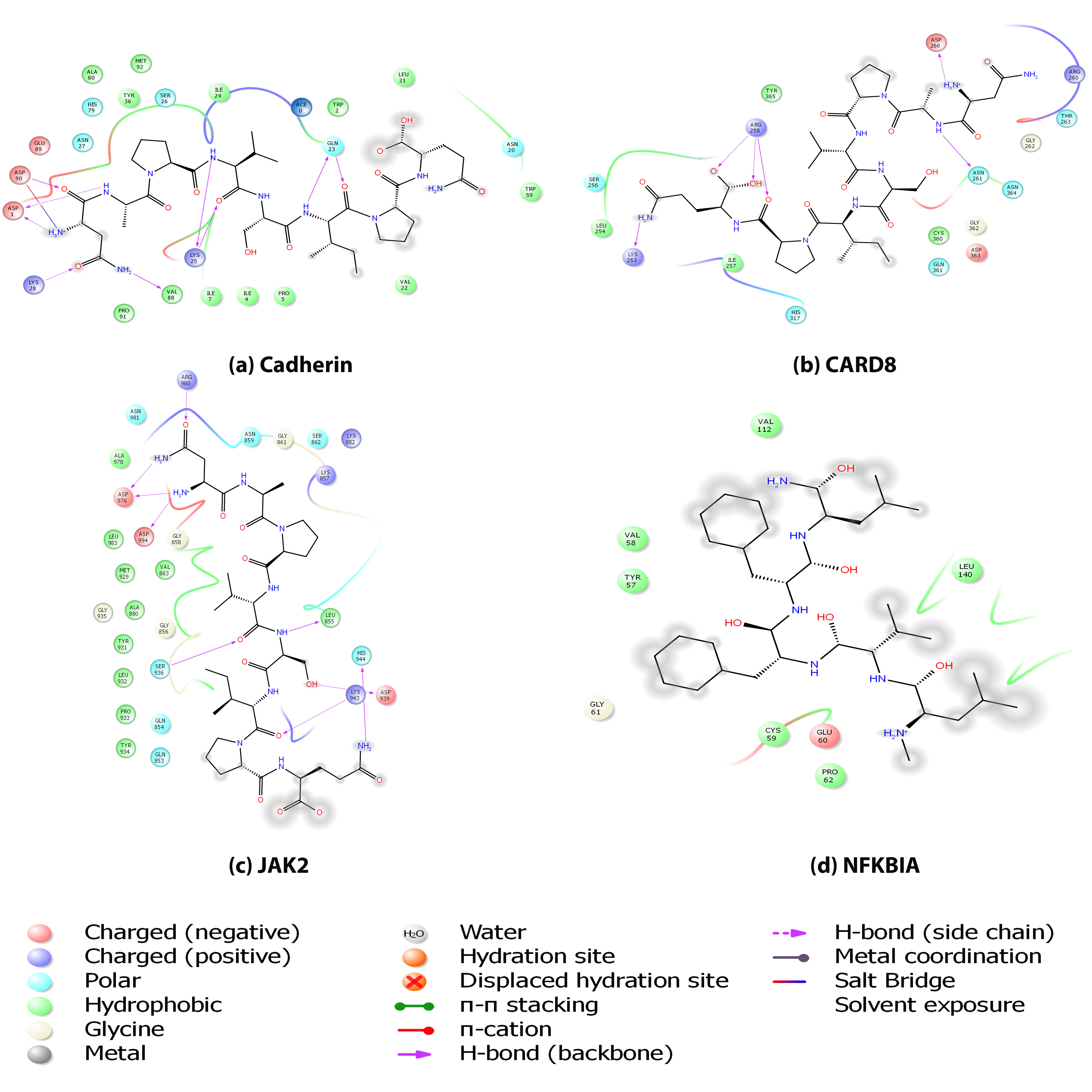

Supplement: Additional file 10: Figure S4. — Shows the post-MD simulation interaction patters of the ligands with the residues of the binding sites of proteins, Cadherin, CARD8, JAK2 and NFKBIA. [file 12864_2016_3108_MOESM10_ESM.jpg]

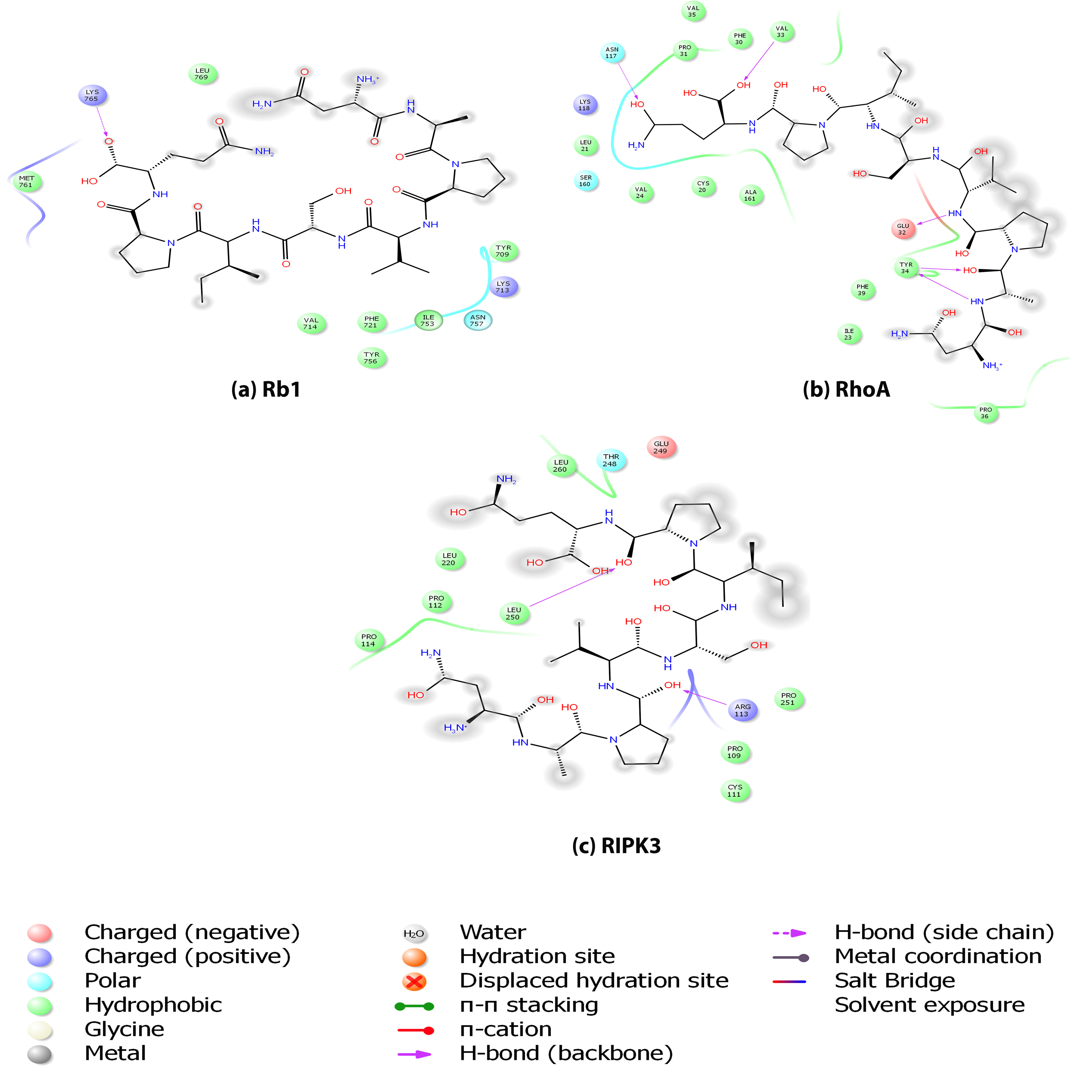

Supplement: Additional file 11: Figure S5. — Shows the post-MD simulation interaction patters of the ligands with the residues of the binding sites of proteins, Rb1, RhoA and RIPK3. [file 12864_2016_3108_MOESM11_ESM.jpg]
